# Body Composition and Survival in Locally Advanced Rectal Cancer Patients Treated with Neoadjuvant Radiochemotherapy

**DOI:** 10.3390/nu17203309

**Published:** 2025-10-21

**Authors:** Piotr Kolenda, Marcin Mardas, Piotr Radomyski, Maciej Trojanowski, Maria Litwiniuk, Wojciech Warchoł, Marta Stelmach-Mardas

**Affiliations:** 1 Greater Poland Cancer Centre, Department of Oncology and Immuno-Oncology, Garbary Str. 15, 61-866 Poznan, Polandmaria.litwiniuk@ump.edu.pl (M.L.); 2 Poznan University of Medical Sciences, Institute of Oncology, Department of Gynaecological Oncology, Szamarzewskiego Str. 82/84, 60-569 Poznan, Poland; 3 Poznan University of Medical Sciences, Electroradiology Department, Garbary Str. 15, 61-866 Poznan, Poland; 4 Greater Poland Cancer Centre, Radiology Department, ul. Garbary 15, 61-866 Poznan, Poland; 5 Greater Poland Cancer Centre, Greater Poland Cancer Registry, Garbary Str 15, 61-866 Poznan, Poland; 6 Poznan University of Medical Sciences, Department of Pathology and Cancer Prevention, Garbary Str. 15, 61-866 Poznan, Poland; 7 Poznan University of Medical Sciences, Department of Optometry, Rokietnicka Str. 5D, 60-806 Poznan, Poland; wwarchol@ump.edu.pl; 8 Poznan University of Medical Sciences, Department of Obesity Treatment, Metabolic Diseases and Clinical Dietetics, Przybyszewskiego Str. 49, 60-355 Poznan, Poland

**Keywords:** rectal cancer, radiochemotherapy, nutritional status, muscle mass, fat mass

## Abstract

**Background:** Nutritional status is a recognized determinant of treatment tolerance and clinical outcomes in oncology. This study aimed to assess body composition using computed tomography (CT) and to evaluate its association with progression-free survival (PFS) and overall survival (OS) in patients with locally advanced rectal cancer (LARC) undergoing curative multimodal therapy. **Methods:** A total of 216 patients with LARC who underwent neoadjuvant chemoradiotherapy (CRT) were retrospectively assessed. Two radiochemotherapy protocols were used: long-course chemoradiotherapy (lcCRT) (radiation therapy administered daily at doses of 1.8 or 2.0 Gy, for a total dose of 50.4–55.8 Gy) with concurrent chemotherapy: either 5-FU with leucovorin or capecitabine and total neoadjuvant chemoradiotherapy (tnCRT)—short-course radiotherapy (5 × 5 Gy) followed by sequential chemotherapy with CAPOX or FOLFOX. Surgery was performed 6.5 weeks after completing CRT. Radiotherapy was delivered using linear accelerators based on the Intensity-Modulated Radiation Therapy technique. CT scans were used to assess nutritional status. Survival analyses were performed. Data on food consumption frequency were collected using the Dietary Habits and Nutrition Beliefs Questionnaire (KomPAN^®^). Non-Healthy-Diet-Index-14 (nHDI-14) was calculated. **Results:** Median observation time was 58 months (range 4–118 months). VATI level and OS (HR: 0.4618 95% CI: 0.2194–0.9719, *p* = 0.0419), as well as SATI and OS (HR: 0.4707 95% CI: 0.2286–0.9693, *p* = 0.0409) were significantly associated. This association was not significant for PFS (VATI: HR: 0.7084 95% CI: 0.4055–1.2376, *p* = 0.2259; SATI: HR: 0.6864 95% CI: 0.3932–1.1981, *p* = 0.1855). SMI and PMI values were not significantly related either PFS (SMI-HR: 0.6728, 95% CI: 0.4031–1.1231, *p* = 0.1295; PMI-HR: 0.7385, 95% CI: 0.4628–1.1785, *p* = 0.2036) or OS (SMI-HR: 0.9128, 95% CI: 0.4703–1.7720, *p* = 0.7876; PMI-HR: 0.6592 95% CI: 0.3684–1.1794, *p* = 0.1603). No significant association was found between sarcopenia development and PFS (HR: 1.2733 CI: 0.7589–2.1363; *p* = 0.3602) or OS (HR: 1.1207; CI: 0.5681–2.2107; *p* = 0.7424). Significant differences between men and women in alcohol intake and nHDI-14 were observed. **Conclusions:** Low visceral and subcutaneous adipose tissue index were significantly associated with worse OS in patients with LARC undergoing multimodal treatment. The nHDI-14 was negatively correlated with the duration of observation and patients’ age.

## 1. Introduction

Rectal cancer is a significant public health problem in developed countries, with notable variations in incidence, mortality and survival rates across different regions and genders [1]. The World Age-Standardized Rate (ASR) is 7.0 per 100,000 population, where 5561 new cases of rectal cancer were registered in Poland in 2021 [2]. A gender-specific analysis shows that rectal cancer is more prevalent among men [3]. According to the TNM Classification of Malignant Tumours, the majority of patients are diagnosed at advanced stages (III, IV) [1]. Such a clinical situation of patients has spurred investigations into the use of total neoadjuvant therapy, including induction or consolidation chemotherapy, and a shift towards treatment protocols that emphasize organ preservation [2].

The nutritional status of oncological patients plays an important role in the therapeutic outcomes, including longer overall survival (OS), and the patient’s quality of life (QoL). Sarcopenia is currently one of the most extensively studied body composition phenotypes, identified as a novel radiological biomarker predicting poorer survival in predominantly colon cancer cohorts [3]. Nevertheless, much less is known about the direct impact of sarcopenia on treatment and survival in patients with stage III—locally advanced rectal cancer (LARC) [4]. In particular, data regarding the Caucasian population are limited. A few retrospective studies have linked sarcopenia with OS in the Asian cohort [5,6]. Malnutrition and changes in nutritional status are not only observed after chemoradiotherapy (CRT) but are also associated with treatment completion and adverse events [7]. Indeed, CRT increases the risk of nutritional deficiencies: undernourished patients with locally advanced rectal cancer more often required delayed treatment and exhibited earlier onset and prolonged duration of nausea, vomiting, and diarrhea compared with well-nourished patients [8]. Therefore, incorporating nutritional assessment into clinical practice is essential for optimizing treatment outcomes.

The aim of this study was to assess body composition with the use of Computed Tomography (CT) scans, including sarcopenia development, and its impact on progression-free survival (PFS) and OS in LARC patients undergoing multimodal treatment with curative intent.

## 2. Materials and Methods

### 2.1. Study Design and Study Population

This retrospective observational study involved 216 patients with LARC who underwent neoadjuvant CRT between 2016 and 2022 at the Greater Poland Cancer Centre (GPCC). The study protocol was approved by the Bioethical Committee at Poznan University of Medical Sciences (No KB-80/24; 24 January 2024). The study was carried out in accordance with the Declaration of Helsinki.

The inclusion criteria followed the application of the two radiochemotherapy protocols during the study period: clinical tumor [cT] stage cT4, extramural vascular invasion, clinical nodal [cN] stage cN1 or higher and involved mesorectal fascia. Radiotherapy was performed using linear accelerators based on the Intensity-Modulated Radiation Therapy (IMRT) technique, which allows precise radiation doses to a tumor while minimizing exposure to surrounding healthy tissues. The radiation field included the primary tumor and regional lymph nodes—such as mesorectal, presacral, and internal iliac nodes with obturator or inguinal nodes included in selected cases. A small margin was added to account for movement and setup variability, while organs at risk (small bowel, bladder, and pelvic bones) were carefully spared. The first protocol involved long-course chemoradiotherapy (lcCRT), where radiation therapy was administered daily at doses of 1.8 or 2.0 Gy, for a total dose ranging from 50.4 to 55.8 Gy, depending on the disease stage. In addition, concurrent chemotherapy consisted of either 5-FU with leucovorin or capecitabine regimen chemotherapy. The second protocol was total neoadjuvant chemoradiotherapy (tnCRT)—short-course radiotherapy (5 × 5 Gy) followed by sequential chemotherapy with CAPOX or FOLFOX (according to the RAPIDO protocol) [9]. On average, surgery was performed 6.5 weeks after completing CRT. The adjuvant chemotherapy was permitted in both types of treatment. Exclusion criteria: presence of metastatic disease, recurrent rectal cancer, incomplete documentation, partial treatment or follow up in the GPCC.

### 2.2. Methods

All CT were performed in the GPCC using either GE Optima CT660 (GE Healthcare, Milwaukee, WI, USA) or Siemens Somatom Definition AS scanners (Siemens Healthineers, Erlangen, Germany), typically one month before radiotherapy. Both scanners produced axial images with 1.25 mm slice thickness and a soft tissue reconstruction kernel. To standardize image processing, only non-contrast CT scans performed as part of radiotherapy planning were analyzed. To measure sarcopenia, a single slice from each CT scan, at the level of the transverse processes of the third lumbar vertebra, was selected with the use of the Siemens syngo.via software. Semi-automated software (Coreslicer, accessed on 20 June 2025; available online: https://coreslicer.com/) was applied to measure total skeletal muscle surface area (cm^2^) based on tissue-specific attenuation thresholds. The Hounsfield unit (HU) range for skeletal muscle was set from −29 to +150 HU. The measured muscle groups included psoas major, quadratus lumborum, erector spinae, transversus abdominis, internal and external obliques, and rectus abdominis. The following formulas were used to calculate the Psoas Muscle Index: PMA (cm^2^)/Height (m^2^); subcutaneous adipose tissue index (SATI): SF (cm^2^)/Height^2^ (m^2^) and visceral adipose tissue index (VATI): =VF (cm^2^)/Height^2^ (m^2^) and the Skeletal Muscle Index (SMI): TM (cm^2^)/Height (m^2^). Sarcopenia was defined using sex-specific cut-off points for the L3 Skeletal Muscle Index (SMI), set at 52.4 cm^2^/m^2^ for men and 38.5 cm^2^/m^2^ for women [10,11,12].

The KomPAN^®^ questionnaire was used to assess the dietary patterns in the study population. Available patients were contacted by phone and answered the questions. The food items covered selected products that can be considered as potential dietary risk factors for colon cancer development, such as red meat, fast food, processed meat, alcohol or tobacco use. For each food group, the patient indicated the frequency of consumption within the following ranges: never (0.0), 1–3 times a month (0.06), once a week (0.14), several times a week (0.5), once a day (1.0), and several times a day (2.0) [13]. The Non-Healthy Diet Index-14 (nHDI-14) was calculated by summing the daily frequencies of selected food group consumption (times/day). The nHDI-14 included food items indicating less healthy foods, for example white bread; fast foods; fried foods; smoked sausages; and red meat; or sweets, where the score ranged between 0 and 28 points, with further standardization to a 0–100-point scale for the diet quality score calculation [13].

### 2.3. Statistical Analysis

If a non-Gaussian distribution was revealed by the D’Agostino and Pearson test, the data are presented as median and range. If a normal distribution was confirmed, the mean ± standard deviation is shown. The *t*-test was used to compare normally distributed variables; otherwise, the Mann–Whitney U test was applied. Comparisons between categorical variables were tested with the Chi-square test. Correlations were tested with the Pearson coefficient for Gaussian distributed data or Spearman for nonparametric data. Correlations were presented as a scatter diagram with trend line (LOESS smoothing span 90%) and heat map. Survival analysis was performed using Kaplan–Meier plots and Cox proportional hazards regression. Data were adjusted for age, sex, BMI, radiotherapy dose, and treatment regimen. SMI at the L3 level of <42 cm^2^/m^2^ in men and <38 cm^2^/m^2^ in women and/or a PMI of <6.36 cm^2^/m^2^ in men and <3.92 cm^2^/m^2^ in women supported a diagnosis of sarcopenia [14]. Cutoff levels for SATI and VATI were assessed using ROC curves with respect to progression status at 3 years of observation. SATI was recognized as low < 36.4 cm^2^/m^2^ in men and <38.6 cm^2^/m^2^ in women (AUC 0.55 sensitivity 44 and specificity 77). Similarly, VATI was recognized as low < 67.3 cm^2^/m^2^ in men and <42.9 cm^2^/m^2^ in women (AUC 0.54, sensitivity 40, and specificity 78). The two tailed *p* value at 0.05 significance level was used for all comparisons. The statistical analyses were computed in Statistica version 13, 2017 (TIBCO Software Inc., 3307 Hillview Avenue Palo Alto, CA 94304, USA) and MedCalc Software Ltd. v23.3.7 (Acacialaan 22, 8400 Ostend, Belgium).

## 3. Results

The clinical characteristics of patients with LARC who underwent CRT are presented in Table 1. The majority of patients were men, with an average age over 60, more than one-third had a normal Body Mass Index (BMI), while over 60% were overweight or obese. The majority had a moderate risk of malnutrition based on their NRS. The largest group consisted of patients with T3 according to TNM classification, N2 and long-course treatment. Significant differences in the analyzed CT parameters were observed between women and men. Gender did not influence the differences in BMI values (Table 2). The database cutoff date was made on 30 June 2025. Median observation time was 58 months (range 4–118 months). During the observation time, progression was diagnosed in 90 patients, and 53 deaths were reported.

**Figure 1:**
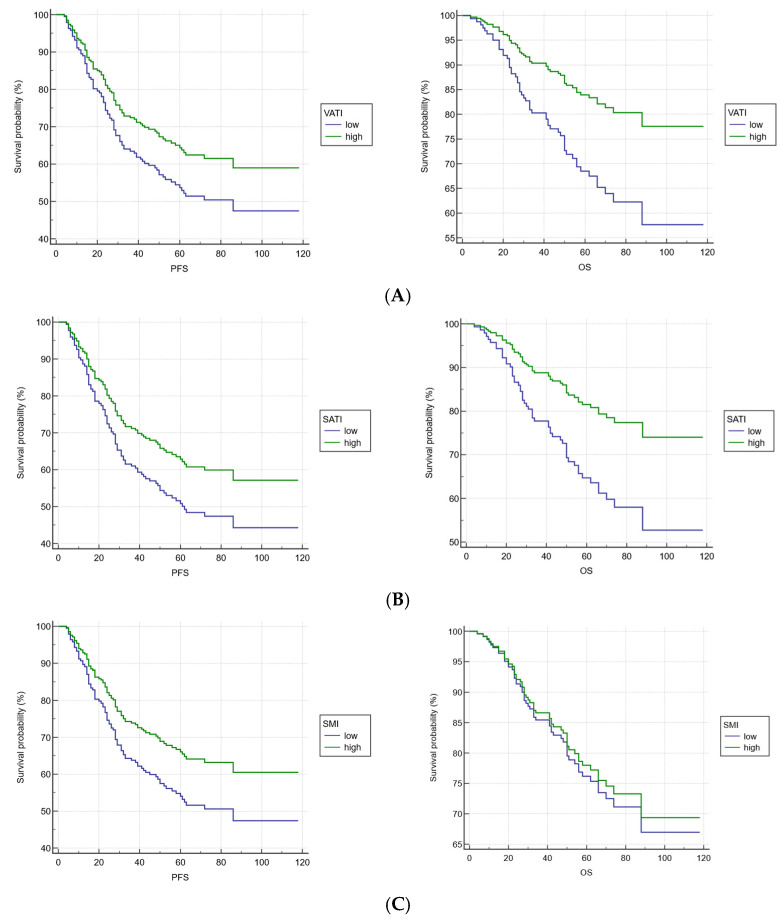

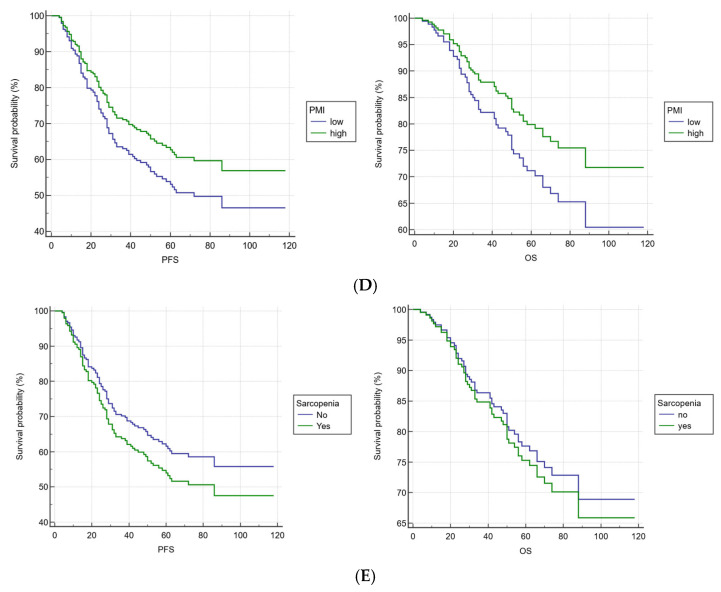
Kaplan–Meier plots for survival in locally advanced rectal cancer (LARC) patients who underwent the neoadjuvant chemoradiotherapy based on (**A**) visceral adipose tissue level (VATI); (**B**) subcutaneous adipose tissue level (SATI); (**C**) Skeletal Muscle Index (SMI); (**D**) Psoas Muscle Index (PMI); (**E**) sarcopenia development; data adjusted for age, sex, Body Mass Index (BMI), radiotherapy dose, treatment regimen.

Figure 1 presents Kaplan–Meier plots for PFS and OS with regard to VATI, SATI, SMI, PMI and sarcopenia. Due to good prognosis, the median survival is not yet reached in the study population, so the median survival is not reported. However, when adjusting for age, sex, Body Mass Index (BMI), radiotherapy dose, and treatment regimen, a significant association was observed between VATI level and OS (HR: 0.4618 95% CI: 0.2194–0.9719, *p* = 0.0419), as well as between SATI and OS (HR: 0.4707 95% CI: 0.2286–0.9693, *p* = 0.0409). Low VATI and SATI levels were associated with worse OS in this group of patients. This observation was not significantly confirmed in PFS models (VATI: HR: 0.7084 95% CI: 0.4055–1.2376, *p* = 0.2259; SATI: HR: 0.6864 95% CI: 0.3932–1.1981 *p* = 0.1855). Separate models for SMI and PMI were created to determine adjusted hazard ratios (HR) for these patients, but no significant results were obtained for either PFS (SMI-HR: 0.6728, 95% CI: 0.4031–1.1231, *p* = 0.1295; PMI-HR: 0.7385, 95% CI: 0.4628–1.1785, *p* = 0.2036) or OS (SMI-HR: 0.9128, 95% CI: 0.4703–1.7720, *p* = 0.7876; PMI-HR: 0.6592 95% CI: 0.3684–1.1794, *p* = 0.1603). No significant association was found between sarcopenia development and PFS (HR: 1.2733 CI: 0.7589–2.1363; *p* = 0.3602) or OS (HR: 1.1207; CI: 0.5681–2.2107; *p* = 0.7424) (Table 3).

**Figure 2:**
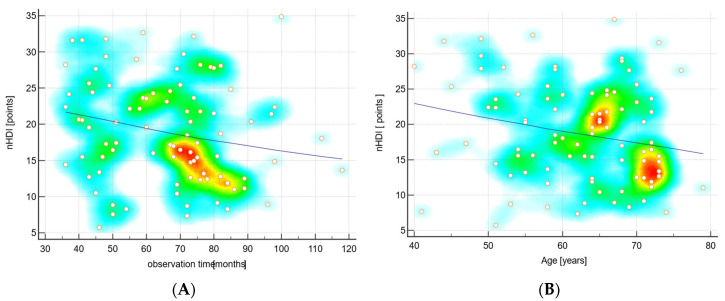
Scatter diagrams with trend line (LOESS smoothing span 90%) and heat map for correlations: (**A**) non-Healthy Diet Index (nHDI) and observation time (*p* < 0.05); (**B**) nHDI and age (*p* < 0.05).

Of all study participants, 92 agreed to answer questions regarding the frequency of selected food consumption. The data indicated differences between men and women in alcohol intake (Table 4). nHDI-14 scores were generally low in the study population, although to a greater extent in men than in women. The nHDI-14 was significantly associated with the duration of observation and patients’ age. The longer a patient was observed and the older he was, the lower his nHDI-14 score (Figure 2).

## 4. Discussion

The study confirmed a clear association between body composition parameters and survival in patients with locally advanced colorectal cancer undergoing multimodal treatment with curative intent. Low visceral and subcutaneous adipose tissue significantly worsened overall survival. These observations are valuable and should be of interest to clinicians, since initial assessment based on BMI alone may not indicate malnutrition, whereas objective imaging methods allow a more accurate evaluation of nutritional status. CT scans are routinely used to monitor disease progression. Considering these core benefits, nutritional assessment using the same CT scans should not be viewed as an additional obligation but rather as an essential component of clinical practice. Indeed, better-nourished patients demonstrate improved responses to treatment.

Treatment of rectal cancer patients is complex, and each intervention, whether radiochemotherapy or chemotherapy, carries potential complications. According to the analysis by Ghalehtaki et al. [15], no definitive conclusion could be drawn regarding the ideal sequencing of chemotherapy, radiotherapy, or combined modalities. Induction chemotherapy offers the advantage of early intervention against micrometastatic disease, whereas consolidation chemotherapy following radiotherapy is associated with higher rates of pathological complete response (pCR) and may be particularly beneficial for patients with inoperable tumors, also contributing to symptom relief. Therefore, an individual therapeutic approach to oncological patients is crucial. Moreover, data indicate that good nutritional status and proper body composition are essential to achieve optimal therapeutic outcomes and to improve patients’ quality of life by reducing treatment-related side effects [16]. Various parameters can be measured in CT to assess changes in body composition. Sometimes, depending on the studied population, certain parameters may emerge as potential predictors of OS assessment, further emphasizing the importance of integrating body composition analysis into routine CT evaluation of cancer patients.

According to Yamato et al. [7], alterations in nutritional status frequently occur during radiochemotherapy and are significantly associated with increased incidence of adverse events and early discontinuation of treatment. In the current study, VATI and SATI were identified as predictors of OS. Li et al. [17] identified a novel prognostic factor linked to the development of adverse events during total neoadjuvant radiochemotherapy and postoperative complications in LARC patients. Using CT-based assessments, high-risk patients were characterized as those with low baseline visceral adipose (≤29.5 cm^2^) and experienced a reduction in visceral adipose during chemoradiotherapy, as these individuals were more likely to encounter adverse events. Conversely, an increase in adipose tissue was associated with a lower incidence of complications. Notably, any change in visceral fat—whether loss or gain—among patients with visceral obesity (≥105 cm^2^) correlated with higher rates of perioperative complications. Visceral obesity has also been identified as a negative prognostic factor in patients with resectable LARC undergoing primary surgical resection, whereas an increase in gluteal subcutaneous adipose during neoadjuvant chemoradiotherapy may be indicative of more favorable clinical outcomes [18]. Bocca et al. [19] demonstrated that a higher ratio of visceral to subcutaneous adipose is strongly correlated with an elevated risk of postoperative complications and prolonged hospitalization in patients with advanced rectal cancer undergoing surgical treatment. Clark et al. [20] showed that elevated visceral adiposity was linked to an increased recurrence risk, particularly in patients with well-to-moderately differentiated tumors and those with incomplete response to neoadjuvant chemoradiation treatment. These findings underscore the importance of individualized management and support the development of structured approaches to nutritional assessment and targeted interventions. Nattenmüller’s research [21] drew attention to the wrongly described obesity paradox in cancer patients, where CT-based body composition assessment offers insights that go beyond BMI in explaining this paradox. This is also relevant for our study population, where most patients were overweight or obese according to BMI. On the one hand, an obesity paradox with a protective effect of CT-quantified adipose tissue can be observed for anastomotic leakage and overall surgical complications [21]. On the other hand, high adipose tissue can be associated with higher risk of other surgical complications (wound infections, bladder dysfunction and burst abdomen) and cardiac complications [21]. Clinically, sarcopenic obesity—characterized by excess adiposity combined with reduced skeletal muscle mass—is frequently encountered in oncological patients. Finally, sarcopenia is negatively associated with OS in locally advanced rectal cancer patients treated with neoadjuvant chemoradiation therapy and curative resection while visceral obesity tends to shorten DFS [6]. Alternative approaches to CT data analysis have also revealed that a low subcutaneous/visceral fat ratio correlates with more local postsurgical complications in patients with rectal cancer, while a high intermuscular/subcutaneous fat ratio seems to be associated with worse survival and oncological outcomes [22]. Luo et al. [23] further demonstrated that the visceral-to-subcutaneous adipose tissue ratio measured by CT images is an independent predictor of postoperative anastomotic leakage in rectal cancer patients—acting as a protective factor in males but a risk factor in females. The cited studies highlight the importance of continued research in this area. Each homogeneous group of patients assessed objectively adds valuable information to clinical practice. We can assume that the data and observations obtained in the assessment of patients’ body composition may differ because they were made using different software. Nevertheless, the studies by van Vugt et al. [24] indicated consistency in measurements of cross-sectional muscle area (CSMA), VATI, and SATI on abdominal CT scans, enabling reliable comparisons across studies. Quantitative evaluation of skeletal muscle mass and adipose tissue distributions at initial diagnosis were important predictors of long-term oncological outcomes in rectal cancer patients [25]. Skeletal muscle radiodensity (SMD) and skeletal muscle mass index (SMI) were independent factors for predicting OS, while SMD and mesorectal fat area (MFA) were independent factors for predicting disease-free survival (DFS) in patients with middle and low rectal cancer undergoing radical surgery [25]. Temel et al. [26] confirmed that mesorectal adipose tissue volume and VAT are useful prognostic markers in rectal cancer. An exploratory study on surgically treated rectal cancer patients showed that skeletal muscle loss, after neoadjuvant chemoradiotherapy, negatively impacts DFS [27]. Levolger et al. [28] confirmed that skeletal muscle loss during neoadjuvant chemoradiotherapy in rectal cancer patients is an independent prognostic factor for DFS and distant metastasis-free survival following curative intent resection. Wei et al. [29] applied AI-based technology to measure the body composition of the entire lumbosacral (L1-S5) region in rectal cancer patients treated with neoadjuvant therapy followed by surgery, again confirming skeletal muscle (SM) as a key component of body composition in this group of patients. Additionally, an intramuscular adipose tissue (IMAT) seems to be a potential player in tumor immunology [28]. Finally, Chilorio et al. [30] suggested that the assessment of muscle and fat mass may support the improved management of LARC patients undergoing RT.

We have to highlight that nutritional status is directly related to diet. Several dietary factors have been associated with the risk of colorectal cancer (CRC). Mols et al. [31], based on data from the Norwegian Women and Cancer study, indicated a significant correlation between the intake of ultra-processed foods (UPF) and the risk of CRC. El Kinany et al. [32] confirmed these findings in a Moroccan population. Interestingly, a study by S. Deoula et al. [33], conducted in the same population, investigated red and white meat subtypes and processed meats (divided into traditional “Khlii, Kaddid” and industrially processed meat). The authors demonstrated not only similar associations between red meat consumption and CRC risk in Morocco as in developed countries, but also inverse associations for traditionally processed meat products. This suggests that the effects of traditional versus Western-style processed meat consumption in developing countries may differ. An analysis of data from the NIH-AARP Diet and Health Study cohort [34] highlighted the importance of whole grains as a source of dietary fiber in CRC prevention. Various dietary scores and indexes (FFQ-based assessments) have been developed by researchers from different regions of the world, usually considering similar dietary components [35]. The nHDI-14 is based on the model of the Polish diet [13].

A previous study conducted in Polish patients [16] showed that their dietary habits changed in a pro-healthy direction, particularly among those undergoing subsequent-line chemotherapy. Women receiving subsequent-line chemotherapy consumed rye bread, pasta, buttermilk, vegetables, fruits, oils, nuts, and juices more frequently, whereas women undergoing first-line chemotherapy consumed more milk, cottage cheese, cream, eggs, fish and seafood, meat offal, salty snacks, and jam. In the current study, patients reduced their intake of non-healthy foods over the observation period, which was reflected by a lower nHDI-14 intensity. This finding highlights the importance of education at both stages—prevention and treatment. Future directions of investigation should focus on targeted dietary interventions in patients with low VATI/SATI, further search for an association between the nHDI14 index with CT-derived body composition parameters. There is different software available on the market that can be used for nutritional status assessment. For example, ODIASP that combines two algorithms to automate L3 slice selection and skeletal muscle segmentation was presented as a reliable tool [36]. Core slicer and Siemens syngo.via can be also reliable which was also demonstrated in studies previously published by our team [37,38] and in the current study. It seems that the use of good software in the hands of an experienced person does not constitute an advantage of either. Any step forward in the daily use of CT scans in body composition assessment will be an advantage, where an automated report integration would be one way to go. However, recently published studies [39,40,41] have taken radiology to a completely different dimension of thinking about clinical work, where AI is a tool that significantly facilitates work. The fully automated AI-integrated system for Body Composition Assessment on CT was already used and presented as excellent in rapid and accurate screening, which also remains a challenge for radiologists [41].

Among the limitations, we utilized the hospital database of a single clinical center, although the largest one in the Greater Poland Region. Although the overall group was large, there were relatively small groups of patients with low body weight and, consequently, low BMI values. This certainly influenced the obtained sarcopenia values. We analyzed a homogeneous group of patients treated according to the same protocol over the last few years, which is a strength of the study. Due to the single-center nature of this study, the obtained results must be taken with caution, as they may not be generalized to other patient populations. Finally, it should be emphasized that the FFQ was completed by less than 50% of patients, which is due to the specific nature of the oncology patient population. Data could only be obtained from patients who were still alive and willing to cooperate, which does not change the fact that selection bias in the assessment of dietary habits may have occurred.

## 5. Conclusions

In conclusion, low visceral and subcutaneous adipose tissue indices significantly worsened OS in patients with LARC undergoing multimodal treatment with curative intent. These findings suggest that adipose tissue assessment may help identify high-risk patients and support the integration of body composition analysis into routine clinical practice. Although the nHDI-14 was negatively correlated with the duration of observation and patients’ age, it should be noted that the nHDI-14 values were expressed at a low level. This suggests that oncological patients pay more attention to, and are more aware of, proper dietary habits.

## Figures and Tables

**Table 1:** Clinical characteristics of patients with locally advanced rectal cancer undergoing multimodal treatment with curative intent (n = 216).

Study population (no)	216
Females/Males (no)	87/129
(%)	40/60
Age (mean ± SD)	61.2 ± 8.7
TNM classification no (%)	
T2	4 (1.9)
T3	144 (66.7)
T4	68 (31.5)
N0	10 (4.6)
N1	30 (13.9)
N2	174 (80.6)
N3	1 (0.5)
Tumor grade no (%)	
1	30 (13.9)
2	105 (48.6)
3	12 (5.6)
N/A	69 (31.9)
Treatment	
lcCRT	166 (76.9)
tnCRT	50 (23.1)
Body mass kg (mean ± SD)	76.6 ± 15.5
Body Heigh cm (mean ± SD)	169 ± 9.7
BMI kg/m^2^ (mean ± SD)	26.8 ± 4.7
<18.5 no (%)	3 (1.4)
18.5–25	77 (35.6)
25–30	94 (43.5)
>30	42 (19.5)
NRS no (%)	
1–2	191 (88.4)
3–5	25 (11.6)

Abbreviations: BMI—Body Mass Index, TNM—Classification of Malignant Tumours, lcCRT—long-course chemoradiotherapy, tnCRT—total neoadjuvant chemoradiotherapy.

**Table 2:** Nutritional status of patients with locally advanced rectal cancer undergoing multimodal treatment with curative intent (n = 216).

Analyzed Parameter	Female		Male		*p*-Value
	Mean	SD	Mean	SD	
BMI [kg/m^2^]	26.97	4.999	26.65	4.439	0.624
SATI [cm^2^/m^2^]	83.66	41.78	48.68	26.47	<0.0001
VATI [cm^2^/m^2^]	51.20	33.21	64.37	35.38	0.0065
SMI [cm^2^/m^2^]	47.59	7.707	57.03	9.443	<0.0001
PMI [cm^2^/m^2^]	5.38	1.27	7.62	1.97	<0.0001

Abbreviations: BMI—Body Mass Index; SATI—subcutaneous adipose tissue level; VATI—visceral adipose tissue level; SMI—Skeletal Muscle Index; PMI—Psoas Muscle Index.

**Table 3:** Hazard ratios for body composition parameters adjusted for age, sex, Body Mass Index (BMI), radiotherapy dose and treatment regimen.

Analyzed Parameter	HR (95% CI)	*p*-Value	HR (95% CI)	*p*-Value
	PFS		OS	
SMI [cm^2^/m^2^]	0.6728 (0.4031–1.1231)	0.1295	0.9128 (0.4703–1.7720)	0.7876
PMI [cm^2^/m^2^]	0.7385 (0.4628–1.1785)	0.2036	0.6592 (0.3684–1.1794)	0.6592
Sarcopenia [cm^2^/m^2^]	1.2733 (0.7589–2.1363)	0.3602	1.1207 (0.5681–2.2107)	0.7424
VATI [cm^2^/m^2^]	0.7084 (0.4055–1.2376)	0.2259	0.4618 (0.2194–0.9719)	0.0419
SATI [cm^2^/m^2^]	0.6864 (0.932–1.1981)	0.1855	0.4707 (0.2286–0.9693)	0.0409

Abbreviations: SATI—subcutaneous adipose tissue level, VATI—visceral adipose tissue level, SMI—Skeletal Muscle Index, PMI—Psoas Muscle Index, HR—Hazard Ratio, PFS—progression-free survival, OS—overall survival.

**Table 4:** Food consumption frequency in available patients who underwent neoadjuvant chemoradiotherapy (n = 92).

Analyzed Factor	Female (no = 39)	Male (no = 53)	*p*-Value
Meal number no (%)			*p* > 0.05
2–3	11 (28)	26 (49)	
4	15 (38)	14 (26)	
5	13 (34)	13 (25)	
Red meat intake no (%)			*p* > 0.05
Less than once a week	12 (31)	15 (28)	
More than once a week	27 (69)	38 (72)	
Processed meat intake no (%)			*p* > 0.05
Less than once a week	4 (10)	3 (6)	
More than once a week	35 (90)	50 (94)	
Fast food intake no (%)			*p* > 0.05
Less than once a week	39 (100)	51 (96)	
More than once a week	0 (0)	2 (4)	
Sweets intake no (%)			*p* > 0.05
Less than once a week	11 (28)	14 (26)	
More than once a week	28 (72)	39 (74)	
Fruit no (%)			*p* > 0.05
Less than once a week	1 (2.5)	0 (0)	
More than once a week	38 (97.5)	53 (100)	
Vegetables no (%)			*p* > 0.05
Less than once a week	1 (2.5)	2 (4)	
More than once a week	38 (97.5)	51 (96)	
Fish no (%)			0.0723
Less than once a week	17 (44)	13 (25)	
More than once a week	22 (56)	40 (75)	
Eggs no (%)			*p* > 0.05
Less than once a week	4 (10)	8 (15)	
More than once a week	35 (90)	45 (85)	
Sweet drinks no (%)			*p* > 0.05
Less than once a week	36 (92)	44 (83)	
More than once a week	3 (8)	9 (17)	
Alcohol no (%)			0.0043
Less than once a week	39 (100)	43 (81)	
More than once a week	0 (0)	10 (19)	
Tobacco no (%)			*p* > 0.05
No	31 (79)	40 (75)	
Yes	8 (21)	13 (25)	
nHDI mean ± SD	15.9 ± 7.12	21.2 ± 6.26	0.0002
nHDI no (%)			*p* > 0.05
low	39 (100)	52 (98.1)	
moderate	0 (0)	1 (1.9)	
heigh	0 (0)	0 (0)	

Abbreviation: nHDI—non-Healthy Diet Index.

## Data Availability

The datasets generated and analyzed during the current study are not publicly available due to privacy policies and internal regulations regarding patient data from the Greater Poland Cancer Centre. However, the data are available from the corresponding authors upon reasonable request. E-mail: stelmach@ump.edu.pl.
